# First postnatal lactate blood levels on day 1 and outcome of preterm infants with gestational age <29 weeks

**DOI:** 10.3389/fped.2024.1443066

**Published:** 2024-10-03

**Authors:** Stephanie Zipf, Ingmar Fortmann, Christoph Härtel, Oliver Andres, Eric Frieauff, Pia Paul, Anna Häfke, Heiko Reutter, Patrick Morhart, Ursula Weller, Amrei Welp, Henry Kipke, Egbert Herting, Alexander Humberg, Wolfgang Göpel, Kathrin Hanke

**Affiliations:** ^1^ Department of Pediatrics, University of Würzburg, Würzburg, Germany; ^2^ Department of Pediatrics, University Hospital Schleswig-Holstein/ Campus Lübeck, Lübeck, Germany; ^3^ Department of Pediatrics, and Adolescent Medicine, Devision of Neonatology and Pediatric Intensive Care, University Hospital Erlangen, Erlangen, Germany; ^4^ Department of Pediatrics, University of Bielefeld, Bielefeld, Germany; ^5^ Department of Obstetrics and Gynaecology, University Hospital Schleswig-Holstein/Campus Lübeck, Lübeck, Germany; ^6^ Department of Pediatrics, University Hospital Münster, Münster, Germany

**Keywords:** lactate levels, small for gestational age, extremely preterm infants, intraventricular haemorrhage, bronchopulmonary dysplasia, all-cause mortality

## Abstract

**Background:**

Serum lactate levels are used as biomarkers for perinatal asphyxia, while their value for outcome prediction in preterm infants is uncertain. It was the aim of this observational study to determine the association of the first postnatal serum-lactate levels on day 1 of life and short-term outcome in preterm infants less than 29 gestational weeks.

**Methods:**

We analysed data in a population-based cohort of German Neonatal Network (GNN) preterm infants with available first postnatal lactate levels enrolled at 22–28 weeks of gestational age (GA) between 1st of April 2009 and 31st December 2020. We hypothesized that high lactate levels as measured in mmol/L increase the risk of intraventricular haemorrhage (IVH) and bronchopulmonary dysplasia (BPD) in infants with VLBW regardless of small-for-gestational-age (SGA) status. Hypotheses were evaluated in univariate analyses and multiple logistic regression models.

**Results:**

First postnatal lactate levels were available in 2499 infants. The study population had a median GA of 26.7 [IQR 25.2–27.9] weeks and birth weight of 840 g [IQR 665–995]. Infants with short-term complications such as IVH and BPD had higher initial lactate levels than non-affected infants. The positive predictive value of a lactate cut-off of 4 mmol/L was 0.28 for IVH and 0.30 for BPD. After adjustment for known confounding variables, each 1 mmol/L increase of day 1 lactate levels was associated with a modestly increased risk of IVH (OR 1.18; 95% CI 1.03–1.37; *p* = 0.002) and BPD (OR 1.23; 95% CI 1.06–1.43; *p* = 0.005) but not with sepsis or mortality. Notably, SGA was associated with lower risk of any grade and severe IVH (OR 0.70; 95% CI 0.54–0.85; *p* = 0.001).

**Conclusions:**

In our observational cohort study higher initial lactate levels were associated with adverse outcome regardless of SGA status. However, the predictive value of lactate cut-off levels such as 4 mmol/L is low.

## Introduction

Serum lactate has been described as predictive measure for neonatal outcome in certain settings, e.g., hypoxic ischemic encephalopathy after perinatal asphyxia (1). Lactate is produced as a consequence of reduced or critically low oxygen supply, when anaerobic metabolism is necessary to meet the tissues energy requirements. Hence, lactate levels reflect tissue perfusion and oxygenation as well as metabolic capacity at the cellular level. Increased lactate levels may therefore be a consequence of acute intrauterine or perinatal stress. In addition, lactate acidosis may reflect a situation of chronically disturbed supply to the fetus, particularly in the context of intrauterine growth restriction.

High intrapartum lactate levels have been associated with an increased risk of adverse developmental long-term outcome in infants >34 gestational weeks with prenatal pathological cardiotocogram (2). However, the role of lactate as biomarker for the outcome of preterm infants is not well understood. In a recent retrospective study including 254 preterm infants with very-low-birthweight (VLBW, birth weight <1,500 g), postnatal lactate levels measured within the first 72 postnatal hours were higher in infants with less favourable outcome as compared to good outcome, however, the discrimination was poor (3). Others have suggested that base deficit and serum lactate concentrations may be important prognostic indicators, i.e., for the development of intraventricular haemorrhage (IVH) in preterm infants with a mean gestational age (GA) between 25 and 29 weeks (4–7). Notably, the risk of IVH in infants being SGA might be reduced despite higher lactate levels at birth in the context of intrauterine growth restriction (8–10).

Large scale data on lactate levels in the first hours of life and their correlation with outcome in preterm infants with very high vulnerability are missing. We therefore analysed data in a population-based cohort of 12,114 infants with VLBW enrolled in the German Neonatal Network (GNN) at 22–28 weeks of GA. In a subset of 2,499 infants, we performed additional monitoring of first postnatal lactate levels on day 1 of life. We hypothesized that high lactate levels as measured in mmol/L increase the risk of IVH and bronchopulmonary dysplasia (BPD) in infants with a birth weight below 1,500 g regardless of small-for-gestational-age (SGA) status.

## Methods

### Patient population

The German Neonatal Network (GNN; www.vlbw.de) is a multicenter collaboration of 68 tertiary level care neonatal units across Germany to study risks and complications of infants being VLBW born at 22 + 0–36 + 6 weeks of gestation at a population-based level. Main short-term outcomes are assessed at discharge from primary stay in hospital, a subgroup of infants has been followed up until early school age.

For the purpose of this observational study, we analyzed short-term outcome data of infants with a GA of 22 + 0 days to 28 + 6 days born in GNN centers between 1st of April 2009 and 31st December 2020. After obtaining written informed parental consent, enrollment in GNN was performed and 250 predefined parameters were recorded for each patient on clinical record files. After discharge, data sheets were sent to the leading study site (University of Lübeck). Data quality was evaluated by a study team physician trained in neonatology via annual on-site monitoring. In this context, a baseline dataset of eligible, but infants that were not enrolled including SGA status was documented.

### Follow-up

When children participating in the GNN reached 5–6 years of age, the study office invited them for a follow-up evaluation. The contact database was searched randomly to identify potential candidates, prioritizing infants born before 28 weeks of gestation, although those born after 28 weeks were not excluded. No other clinical criteria were used to identify potential candidates. This invitation process was standardized across all participating centers. A single GNN follow-up team conducted the assessments at the sites, unaware of any interventions or complications during the children's initial NICU stay. The follow-up evaluation consisted of hearing tests, visual screenings, and spirometry. Parents provided information about their child's medical history, current health needs, social background, illnesses, and overall development and behavior.

### Definitions

First postnatal lactate levels were determined at the discretion of the attending neonatologist by arterial, venous or capillary blood withdrawal but not cord blood. All blood samples for lactate value evaluation were collected within the first 24 h after birth, whereas the first available lactate value was documented and analyzed. We focused on the first postnatal lactate levels on day 1 of life because these values are most indicative of the immediate perinatal condition and physiological adaptation of the preterm infant. Unreliable data points were excluded (negative values and values >30 mmol/L), ensuring that our analysis reflects an accurate dataset. GA was calculated from the best obstetric estimate based on early prenatal ultrasound and obstetric examination (11). Small for gestational age (SGA) was defined as a birth weight less than the 10th percentile according to gender-specific standards for birth weight by postmenstrual age in Germany (12). IVH was defined as ultrasound diagnosis based on Papile's grading (13, 14). Periventricular leukomalacia (PVL) was defined as periventricular, cystic white matter lesion diagnosed by cranial ultrasound (15). Bronchopulmonary dysplasia (BPD) was defined as need of oxygen or respiratory support [continuous positive airway pressure (CPAP) or mechanical ventilation] at 36 weeks' postmenstrual gestational age. Death was defined as all-cause mortality during the primary stay in hospital. Sepsis was defined according to the criteria of the national infection surveillance system “NEO-KISS” (16). Clinical sepsis was defined as sepsis with at least two clinical signs (temperature >38°C or <36.5°C, tachycardia >200/min, new onset or increased frequency of bradycardias or apneas, hyperglycemia >140 mg/dl, base excess <−10 mval/L, pale/grey skin color, increased oxygen requirements) or one clinical and one laboratory sign (C-reactive protein >1 mg/dl, immature neutrophil/total neutrophil ratio >0.2, white blood cell count <5/nl, platelet count <100/nl) and antibiotic treatment for ≥5 days, but no proof of causative agent in the blood culture. Blood-culture proven sepsis was defined as clinical sepsis with proof of causative agent in the blood culture. If *Coagulase negative staphylococci (CoNS)* were detected as single pathogen in the blood culture, a CrP value of >1 mg/dl was mandatory to be defined as true *CoNS* sepsis. *Early onset sepsis* was defined as blood-culture confirmed sepsis occurring within the first 72 h after birth, *late onset sepsis* was defined as blood-culture confirmed sepsis after 72 h. Necrotizing enterocolitis (NEC) was defined as NEC stage 2 or 3 according to Bell with need for surgical intervention (17). Focal intestinal perforation (FIP) was defined as spontaneous intestinal perforation requiring surgery (18). Retinopathy of prematurity (ROP) was defined as ROP requiring treatment (anti-VEGF, cryo- or laser therapy) (19). The composite outcome of severe complications was defined as either surgery due to NEC or FIP or treatment for ROP or IVH grade III/IV, PVL or death.

### Statistical analysis

Data analyses were performed using SPSS 29.0 (IBM, Munich, Germany). Hypotheses were evaluated in univariate analyses using Chi-square test for categorical variables and Mann-Whitney *U*-test for continuous variables for comparisons. In univariate analyses, we stratified to SGA status, to lactate levels in mmol/L and to infants with a level cutoff of 4 mmol/L (20). We tested our hypothesis for the following outcome parameters as defined above (see definitions): IVH, severe IVH (grade III and IV), sepsis, BPD, death, NEC and a composite outcome of severe complications (surgery due to NEC or FIP or treatment for ROP or IVH grade III/IV, PVL or death).

We conducted 14 multiple logistic regression models to assess the effect of SGA and lactate levels (per mmol/L or >4 mmol/L) on the above-mentioned outcomes, reporting odds ratios and 95% confidence intervals. All models were adjusted for known risk factors of adverse outcomes in preterm infants including GA, sex, multiple birth, delivery mode, outborn delivery, antenatal steroids, Apgar score at 5 min, early-onset sepsis (EOS) and inotrope use in the first 24 h as a surrogate measure for a complicated clinical course. Results for SGA were used from models with lactate level as per mmol/L as dependent variable. For models to assess the lactate variables, SGA was included as confounder. In the model for sepsis, EOS was excluded as a confounder. The Bonferroni-adjusted type I error was accordingly set at 0.004 to account for multiple testing. We used a uniform dataset with available data for all metric parameters. Missing data were not imputed.

### Ethics

The study was conducted according to the guidelines of the Declaration of Helsinki and approved by the Ethical Committee of the University of Lübeck, as well as the committees of the participating centers (vote no. 08-022, date of approval: June 27th, 2008).

This report followed the STROBE guidelines for reporting data of observational cohort studies (21).

## Results

### Clinical characteristics of the study cohort

A total of *n* = 12,114 infants with a GA of 22–28 weeks were included in the GNN study, of which 1,582 (12.6%) were SGA. Mean GA was 26.9 weeks for infants not being SGA vs. 25.7 weeks for the infants with SGA status, while mean birth weight differed accordingly (895 vs. 490 g). In a subset of 2,499 infants with a mean GA of 26.7 weeks and birth weight of 840 g we documented first postnatal lactate levels (Table 1). This subset did not remarkably differ in clinical characteristics from all other infants that we enrolled in the GNN (Supplementary Table S1).

**Table 1:** Clinical characteristics of preterm infants stratified to SGA status.

Clinical characteristics	>10th perc	SGA	Total
Number of infants	2,183	316	2,499
GA (weeks) [median (IQR)]	26.9* [25.3–27.9]	25.9* [24.4–27]	26.7 [25.2–27.9]
Birth weight (g) [median (IQR)]	880* [730–1,050]	495* [460–575]	840 [665–995]
APGAR score 5 min [median (IQR)]	8 [7–8]	7 [6–8]	7 [6–8]
APGAR score 10 min [median (IQR)]	9* [8–9]	8* [8–9]	9 [8–9]
Umbilical artery pH [median (IQR)]	7.35* [7.29–7.39]	7.29* [7.23–7.36]	7.34 [7.28–7.39]
Antenatal steroids administered (%) (95% CI)	92.6 [91.5–93.7]	93.7 [90.6–96.0]	92.8 [91.7–93.7]
German maternal background (%) (95% CI)	70.5 [68.5–72.4]	71.6 [69–74]	70.6 [68.8–72.4]
Sex (%) (95% CI)			
Male	53.4 [51.3–55.5]	55.2 [49.7–60.6]	53.6 [51.7–55.6]
Female	46.6 [44.5–48.7]	44.8 [39.4–50.3]	46.4 [44.4–48.3]
Multiples (%) (95% CI)	33.7** [31.7–35.7]	24.6** [20.1–29.6]	32.5 [30.7–34.4]

SGA, small for gestational age; GA, gestational age; IQR, interquartile range; CI, confidence interval, Perc, percentile. For comparisons with the group of infants without available lactate levels see Supplementary Table S1.

* *p* < 0.05.

** *p* < 0.01.

### Infants born SGA have a higher risk of adverse short-term outcome except for IVH

In univariate analyses, we noted higher rates of adverse short-term outcome in the SGA-group regarding clinical or culture-confirmed sepsis, surgical interventions for NEC, bronchopulmonary dysplasia, death or the composite outcomes of severe complications. Notably, the rates of IVH were lower in infants born SGA including severe IVH (Table 2; Supplementary Table S1).

**Table 2:** Short-term outcomes of study cohort stratified to SGA.

	>10th percentile (*n* = 2,183)	SGA (*n* = 316)	Total (*n* = 2,499)
Sepsis (%, 95% CI)	14.5*** [13.1–16]	25.9*** [21.4–31]	16 [14.6–17.4]
IVH (%, 95% CI)	27.7 [25.8–29.6]	27.2 [22.5–32.3]	27.6 [25.9–29.4]
IVH III-IV (%, 95% CI)	10.1 [8.9–11.4]	8.9 [6.1–12.4]	9.9 [8.8–11.2]
NEC, surgery (%, 95% CI)	3.3** [2.6–4.1]	7.6** [5.1–10.9]	3.8 [3.1–4.6]
BPD (%, 95%CI)	24.2*** [22.4–26]	54.1*** [48.6–59.5]	27.9 [26.2–29.7]
Composite outcome (severe complications)	21.6*** [19.9–23.3]	37.3*** [32.1–42.8]	23.6 [21.9–25.3]
Death (%, 95% CI)	4.5*** [3.7–5.5]	17.4*** [13.5–21.9]	6.2 [5.3–7.2]

SGA, small for gestational age; IVH, intraventricular haemorrhage; NEC, necrotizing enterocolitis; BPD, bronchopulmonary dysplasia; CI, confidence interval. The composite outcome of severe complications includes IVH grade III/IV, periventricular leukomalacia, surgery for NEC, focal intestinal perforation (FIP), retinopathy of prematurity (ROP) or death.

** *p* < 0.01.

*** *p* < 0.001.

### Infants born SGA have higher lactate levels at birth as compared to infants without SGA status

**Figure 1:**
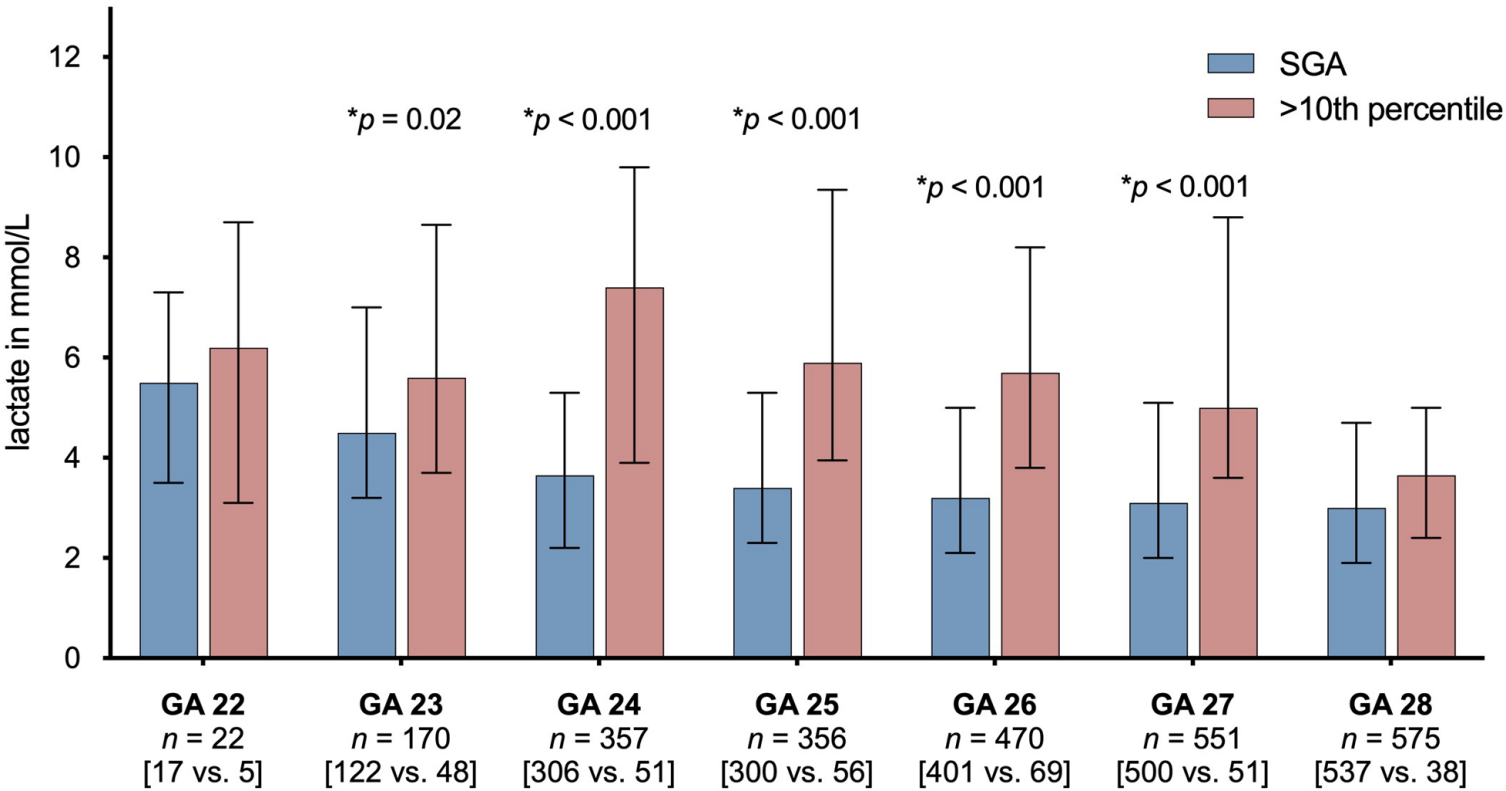
Lactate levels in preterm infants born SGA vs. preterm infants not being SGA stratified to gestational age (GA) subgroups. The figure shows median first postnatal serum lactate levels in mmol/L and interquartile ranges (IQRs) as error bars. *P*-values were derived from Mann-Whitney *U*-test. Only *p*-values, that reached a significant level (<0.05) are displayed. *N* (total) = 2,499 infants. SGA, small for gestational age.

As shown in Figure 1, infants born SGA had higher serum lactate levels than children with a birth weight >10th percentile. These differences were statistically significant for each gestational week strata within the range of 23–27 gestational weeks.

### Higher first postnatal lactate levels are associated with adverse short-term outcomes

In univariate analyses, first postnatal lactate levels were higher in infants who suffered from adverse outcomes (Table 3). We noted median lactate levels of ≥3.8 mmol/L in those infants affected from short-term complications. We calculated test accuracy criteria for level cut-offs of 4 mmol/L to predict IVH [sensitivity 0.84 (95% CI 0.81–0.86), specificity 0.20 (0.18–0.22), positive predictive value 0.28 (0.27–0.29) and negative predictive value 0.76 (0.73–0.80)] or BPD [sensitivity 0.88 (0.85–0.90), specificity 0.22 (0.20–0.24), positive predictive value 0.30 (0.29–0.31) and negative predictive value 0.82 (0.79–0.85)]. Receiver Operating Characteristic (ROC) analyses (curves not shown) showed that the predictive models are marginally above random chance, indicating limited discriminatory power. We observed that the optimal cutoff points from the ROC analysis appear to be approximately 6 mmol/L for IVH and 3 mmol/L for BPD. To account for confounders, we performed multiple logistic regression models adjusting for GA per week, gender, multiple birth, delivery mode, inborn, antenatal steroids, Apgar score at 5 min, early-onset sepsis and inotrope use in the first 24 h. SGA status was associated with lower risk of any grade IVH (OR 0.70; 95% CI 0.54–0.90, *p* = 0.04) and severe IVH (OR 0.70; 95% CI 0.54–0.85; *p* = 0.001). Each 1 mmol/L increase of lactate was associated with increased odds of IVH risk, [1.18 (95% CI 1.03–1.37), *p* = 0.002, Table 4]. The cut-off level of 4 mmol/L, based on a previous report (20), was associated with BPD development but not IVH. Likewise, each 1 mmol/L lactate was associated with a higher risk of BPD [OR 1.23 (95% CI 1.06–1.43), *p* = 0.005].

**Table 3:** Lactate levels in the first hours of life and short-term outcome.

Outcome parameter	Non-affected	Affected	*p*
IVH, any grade	1,819; 3.4 [2.2–5.2]	693; 3.9 [2.4–6.5]	0.001
IVH, grade III/IV	2,262; 3.5 [2.3–5.5]	250; 4.0 [2.4–6.6]	0.076
Sepsis (BC positive)	2,110; 3.5 [2.2–5.5]	400; 3.8 [2.3–6.0]	0.021
Death	2,358; 3.5 [2.2–5.4]	156; 4.7 [2.95–7.6]	<0.001
BPD	1,812; 3.3 [2.1–5.2]	698; 4.0 [2.7–6.5]	<0.001
NEC, surgery	2,418; 3.5 [2.2–5.5]	97; 3.85 [2.75–6.6]	0.45
Composite outcome (severe complications)	1,920; 3.8 [2.2–5.2]	594; 4.0 [2.5–6.8]	<0.001

Data are given as number of infants, median and interquartile range (IQR); BC, blood culture; IVH, intraventricular haemorrhage; BPD, bronchopulmonary dysplasia; NEC, necrotizing enterocolitis. *P*-values were derived from Mann-Whitney *U*-test; the composite outcome of severe complications includes IVH grade III/IV, periventricular leukomalacia, surgery for NEC, focal intestinal perforation (FIP), retinopathy of prematurity (ROP) or death.

**Table 4:** Multiple logistic regression analyses to determine the independent association of SGA and first postnatal lactate levels with short-term outcomes.

Outcome parameter	SGA	Lactate per mmol/L	Lactate >4 mmol/L
IVH (any grade)	OR 0.7 (0.54–0.9), *p* = 0.04 [*n* = 2,327]	OR 1.18 (1.03–1.37), *p* = 0.002 [*n* = 2,327]	OR 1.14 (0.79–1.63), *p* = 0.5 [*n* = 1,216]
IVH (grade III/IV)	OR 0.67 (0.54–0.85), *p* = 0.001 [*n* = 2,327]	OR 1.18 (0.95–1.46), *p* = 0.12 [*n* = 2,327]	OR 0.9 (0.57–1.66), *p* = 0.9 [*n* = 1,216]
Sepsis (BC positive)	OR 1.6 (1.2–2.25), *p* = 0.002 [*n* = 2,325]	OR 0.94 (0.79–1.11), *p* = 0.43 [*n* = 2,325]	OR 0.99 (1.65–1.56), *p* = 0.99 [*n* = 1,216]
Death	OR 3.27 (2.47–3.77), *p* < 0.001 [*n* = 2,329]	OR 0.96 (0.71–1.28), *p* = 0.77 [*n* = 2,329]	OR 0.86 (0.37–2.04), *p* = 0.74 [*n* = 1,217]
BPD	OR 3.08 (2.36–4.04), *p* < 0.001 [*n* = 2,329]	OR 1.23 (1.06–1.43), *p* = 0.005 [*n* = 2,329]	OR 1.57 (1.06–2.31), *p* = 0.023 [*n* = 1,217]
NEC (requiring surgery)	OR 1.18 (0.89–1.56), *p* = 0.24 [*n* = 2,329]	OR 1.35 (0.83–1.56), *p* = 0.4 [*n* = 2,329]	OR 2.11 (0.73–6.0), *p* = 0.1 [*n* = 1,217]
Composite outcome (severe complications)	OR 1.6 (1.41–1.89), *p* < 0.001 [*n* = 2,329]	OR 1.15 (0.98–1.35), *p* = 0.08 [*n* = 2,329]	OR 1.1 (0.74–1.6), *p* = 0.6 [*n* = 1,217]

Data are given as odds ratios (OR), 95% confidence interval (CI), *p*-value and number *n* of cases included in the regression model. BC, blood culture; SGA, small for gestational age; IVH, intraventricular haemorrhage; BPD, bronchopulmonary dysplasia; NEC, necrotizing enterocolitis. All models were adjusted for gestational age per weeks, gender, multiple birth, delivery mode, inborn, antenatal steroids, Apgar score at 5 min, early-onset sepsis, inotrope use in the first 24 h. Results for SGA were used from model with lactate level as per mmol/L. For models to assess the lactate variables, SGA was included as confounder. In the model for sepsis, EOS was excluded as a confounder. The Bonferroni-adjusted type I error was set at 0.004 for to account for multiple testing.

## Discussion

In this observational study on first postnatal lactate levels in 2,499 preterm infants <29 weeks of gestation, we found that each 1 mmol/L increase was associated with an increased risk of IVH or the development of BPD. Cut-off lactate levels of 4 mmol/L for short-term morbidities had low positive predictive values in our setting. Given our data, we conclude that initial lactate levels should be evaluated for future multicomponent risk scores but are not useful as single discriminative biomarker.

It is well known that preterm infants born SGA are at higher risk of complications of preterm birth such as mortality, infections, NEC and BPD (22–24). Preterm infants with intrauterine growth restriction (IUGR) are a worth mentioning portion of infants born SGA. Given the differences in cerebral circulation (brain sparing) and grade of inflammation in preterm infants with intrauterine growth restriction (IUGR) as compared to infants born appropriate-for-gestational-age, the risk of IVH is decreased in the context of SGA (8–10). The association between SGA and a reduced IVH risk was confirmed by our data. On the other hand, the risk of IVH is influenced by a range of complex factors such as gestational age and antenatal steroid exposure (25). In our study, postnatal lactate measurements as potential biomarkers were noted to be elevated in infants being SGA, while increased lactate levels were associated with IVH risk independent of SGA.

Postnatal lactate levels have been shown to be associated with various adverse outcomes in preterm infants. Recent studies evaluating metabolomics technologies in preterm infants <29 weeks of gestation suggest that lactate levels in blood or urine samples may serve as early biomarker of BPD (26, 27). In line with these data, Tuten et al. proposed that serum lactate levels >4 mmol/L are associated with BPD in a small-scale study of 60 infants with VLBW (20). Our data confirm the association between elevated lactate levels and a cutoff of >4 mmol/L with BPD independent of the SGA status.

In contrast to previous studies, we did not find a significant association between early lactate levels on day 1 and either mortality or sepsis, which may be due to various reasons. First, our study specifically focused on preterm infants <29 gestational weeks, whereas previous studies included more heterogeneous neonatal populations with broader GA ranges (4, 5, 20) or different clinical settings where neonatal outcomes, such as sepsis rates, vary significantly, i.e., sepsis rates up to 43% (3) when compared to a total sepsis rate of 16% in our cohort, even including both early and late-onset cases. With more infants being extremely immature, the number of outcome confounders becomes increasingly complex, and more variables may play a dominant role, potentially overshadowing the predictive value of early lactate levels. Second, we specifically analyzed very early lactate levels, which may predict early outcomes, such as early death. However, infants who die very early may be underrepresented in our network due to limited time to obtain informed consent, which may cause selection bias. Finally, there are other studies, which likewise did not find significant associations between lactate levels and sepsis or mortality (20). The conflicting results across studies suggest that elevated lactate levels must be interpreted in the context of other clinical parameters, underlying conditions, and risk factors, which are likely to differ depending on the specific outcome being studied. This variability highlights the importance of considering the broader clinical picture and the interplay of multiple factors when using lactate levels to predict outcomes in neonates. Recent studies have demonstrated that predictive models incorporating multiple variables, including indicators of antioxidant protection, can effectively identify infants at risk of central nervous system injury (28). Similarly, Lee et al. demonstrated that perinatal acidosis is significantly associated with IVH, and that the predictive value can be significantly enhanced when considering additional clinical parameters such as gender, birthweight, and steroid exposure (29). Hence, elevated lactate levels must be interpreted within the broader clinical context, as surrogate measures alone are insufficient for predicting outcomes reliably (30, 31). However, models with high predictive value become increasingly relevant in clinical settings when considering the benefits of preventive therapies, such as prophylactic indomethacin treatment to reduce the IVH risk (32).

Our study is among the largest observational studies with stringent measurements of lactate levels in the first 24 h of life. Our approach has limitations which are inherent in observational studies. First, we only asked attending neonatal teams to document the first postnatal lactate level of the infant which was by arterial, venous or capillary blood withdrawal. Hence, our data are proxy of the supply in the prenatal or immediate perinatal context but cannot provide longitudinal data on the clinical course during the most vulnerable period. Lactate measurements did not follow a standardized approach which might have led to pre-diagnostic and analytical differences. Limited discriminatory power of Receiver Operating Characteristic (ROC) analyses further emphasize that our current findings do not identify a clear cutoff and that further research is required to establish a meaningful threshold. Further, a single lactate measurement is not sufficient to evaluate the potential role of “time to lactate normalization” as a dynamic marker of recovery from a hypoxic situation. Second, although we used regression models to account for possible confounders in our analysis based on observational data, the potential for bias from unknown parameters cannot be completely ruled out, as adjustments cannot fully address all etiological factors of preterm birth, that may influence lactate levels and their link to short-term complications. Further studies are needed to disentangle the intricate relationships between the lactate levels, the metabolome, endogenous risk factors of the infant, nutritional management, and the development of preterm morbidities (33).

## Data Availability

The original contributions presented in the study are included in the article/Supplementary Material, further inquiries can be directed to the corresponding author.
