# Supplementary material for: First postnatal lactate blood levels on day 1 and outcome of preterm infants with gestational age <29 weeks
Source: Front Pediatr. 2024 Oct 3;12:1443066. doi: 10.3389/fped.2024.1443066 (PMC11484003; doi:10.3389/fped.2024.1443066)
Supplement: Supplementary file 1 [file Table1.docx]

**Supplementary material:**

| **Supplementary Table S1.** Clinical characteristics of preterm infants stratified to additional monitoring of lactate (N total=12,114; lactate available=2499, SGA=1266) | | | | | | | |  |
| --- | --- | --- | --- | --- | --- | --- | --- | --- |
|  | **Lactate levels not available** | | | **Lactate levels available** | | | **Total** | |
|  | **>10th perc** | **SGA** | **Total** | **>10th perc** | **SGA** | **Total** |  | |
| **Number of infants** | 8349 | 1266 | 9617 | 2183 | 316 | 2499 | 12,114 | |
| **GA (weeks),**  **median [IQR]** | 26.9*  [25.4 – 28] | 25.7*  [24.4 – 27.3] | 26.7  [25.3 -– 27.9] | 26.9*  [25.2 – 27.8] | 25.9*  [24.4 – 27] | 26.7  [25.1 – 27.9] | 26.7  [25.3 – 27.9] | |
| **Birth weight (g), median [IQR]** | 895*  [735 – 1060] | 490*  [445 – 850] | 850  [668 – 1025] | 880*  [730 – 1050] | 495*  [460 – 575] | 840  [665 – 995] | 846  [665 – 1015] | |
| **APGAR scores 5 min, median [IQR]** | 8  [7 – 8] | 7  [6 – 8] | 8  [7 – 8] | 8  [7 – 8] | 7  [6 – 8] | 8  [7 – 8] | 8  [7 – 8] | |
| **APGAR scores 10 min, median [IQR]** | 9*  [8 – 9] | 8*  [8 – 9] | 9  [8 – 9] | 9*  [8 – 9] | 8*  [8 – 9] | 9  [8 – 9] | 9  [8 – 9] | |
| **Umbilical artery pH, median [IQR]** | 7.34*  [7.29 – 7.38] | 7.30 *  [7.24 – 7.35] | 7.35  [7.28 – 7.38] | 7.35 *  [7.29 – 7.39] | 7.29 *  [7.23 -7.36] | 7.34  [7.28 – 7.39] | 7.34  [7.28 – 7.38] | |
| **Antenatal steroids, % (95%CI)** | 90.9 ***  [90.3 – 91.5] | 94.5 ***  [93.1 – 95.6] | 91.4  [90.8 – 92] | 92.6  [91.5- 93.7] | 93.7  [90.6 – 96] | 92.8  [91.7 -93.7] | 91.7  [91.2 – 92.2] | |
| **German maternal background, % (95%CI)** | 70.8  [69.8 – 71.7] | 71.6  [69 – 74] | 70.9  [70 – 71.8] | 70.5  [71.4 – 70.6] | 71.4  [66.2 – 76.2] | 70.6  [68.8 – 72.4] | 70.8  [70.0 – 71.6] | |
| **Male % (95%CI)** | 53.6  [52.6 – 54.7] | 51.5  [48.8 – 54.3] | 53.4  [52.4 – 54.4] | 53.4  [51.3 – 55.5] | 55.2  [49.7 – 60.6] | 53.6  [51.7 – 55.6] | 53.4  [52.5 -54.3] | |
| **Female % (95%CI)** | 46.4  [54.3 – 47.4] | 48.5  [45.7 – 51.2] | 46.6  [44.5 – 48.7] | 46.6  [44.5 – 48.7] | 44.8  [39.4 – 50.3] | 46.4  [44.4 – 48.3] | 46.6  [45.7 – 47.5] | |
| **Multiples, % (95%CI)** | 34.3***  [33.2 – 35.3] | 20.9***  [18.7 – 23.2] | 32.5  [31.6 – 33.4] | 33.7**  [31.7 – 35.7] | 24.6**  [20.1 – 29.6] | 32.5  [30.7 – 34.4] | 32.5  31.7 – 33.3] | |

***Legend:*** *SGA small for gestational age, GA gestational age, IQR interquartile range, CI confidence interval, perc percentile. * Differences that reach significant levels are given separately for the groups of infants with and without serum lactate values respectively. They relate to the difference between SGA and non-SGA infants in these respective groups. ^*^p<0.05, **p<0.01, ***p<0.001, Chi-square test / Mann-Whitney U test*
